# Invasive pulmonary aspergillosis: role of early diagnosis and surgical treatment in patients with acute leukemia

**DOI:** 10.1186/1476-0711-5-17

**Published:** 2006-07-27

**Authors:** Ridvan Ali, Fahir Ozkalemkas, Tulay Ozcelik, Vildan Ozkocaman, Atilla Ozkan, Sami Bayram, Beyza Ener, Ahmet Ursavas, Guze Ozal, Ahmet Tunali

**Affiliations:** 1 Department of Internal Medicine, Division of Hematology, Uludag University School of Medicine, Bursa, Turkey; 2 Department of Thoracic Surgery, Uludag University School of Medicine, Bursa, Turkey; 3 Department of Microbiology and Infectious Diseases, Uludag University School of Medicine, Bursa, Turkey; 4 Department of Chest and Tuberculosis, Uludag University School of Medicine, Bursa, Turkey

## Abstract

**Background:**

*Aspergillus *is a ubiquitous soil-dwelling fungus known to cause significant pulmonary infection in immunocompromised patients. The incidence of aspergillosis has increased during the past two decades and is a frequently lethal complication of acute leukemia patients that occurs following both chemotherapy and bone marrow transplantation. The diagnosis of invasive pulmonary aspergillosis (IPA) according to the criteria that are established by European Organization for the Research and Treatment of Cancer and Mycoses Study Group raise difficulties in severely ill patients. Despite established improvements in field of diagnosis (galactomannan antigen, quantitative PCR, real-time PCR for *Aspergillus *spp., and findings of computed tomography) and treatment with new antifungals, it is still a major problem in patients with acute leukemia. However, prompt and effective treatment of IPA is crucial because most patients will need subsequent chemotherapy for underlying hematologic disease as soon as possible.

**Case presentation:**

We report a 33-year-old male patient with acute promyelocytic leukemia diagnosed in 1993 that developed invasive pulmonary aspergillosis due to *A. flavus *at relapse in 2003. The patient was successfully treated with liposomal amphotericin B and underwent surgical pulmonary resection. The operative course was uneventful.

**Conclusion:**

This report emphasizes the clinical picture, applicability of recent advances in diagnostic and therapeutic approaches for IPA. For early identification of a patient infected with IPA, a high index of suspicion and careful clinical and radiological examinations with serial screening for galactomannan should be established. If aspergillosis is suspected, anti-aspergillosis drug should be administered immediately, and if a unique pulmonary lesion remains, surgical resection should be considered to prevent reactivation during consecutive chemotherapy courses and to improve the outcome.

## Background

*Aspergillus *is a ubiquitous soil-dwelling fungus known to cause significant pulmonary infection in immunocompromised patients. The incidence of aspergillosis has increased during the past two decades due to widespread use of chemotherapy and immunosuppressive agents, and is a frequently lethal complication of acute leukemia patients that occurs following both chemotherapy and bone marrow transplantation (BMT) [1]. The mortality rate reaches 50% for chemotherapy-induced neutropenia and can exceed 90% in BMT patients [2,3]. Early diagnosis and treatment of infection are crucial, however, definite diagnosis is not straight-forward and requires invasive procedures that are often difficult or impossible due to bleeding tendency or poor condition of patient, and the diagnostic yield of bronchoalveolar lavage, including cytology and fungal culture, is low. Despite established improvements in field of diagnosis (galactomannan antigen (GM), quantitative PCR, real-time PCR for *Aspergillus *spp., and findings of computed tomography) and treatment, it is still a major problem in patients with acute leukemia [4-6]. Recently, because of the difficulties in diagnosis of IPA, the European Organization for Research and Treatment of Cancer (EORTC) Invasive Fungal Infections Cooperative Group and the Mycoses Study Group (MSG) of the National Institute of Allergy and Infectious Disease established consensus definitions for defining opportunistic invasive fungal infections [7]. Although, the EORTC/MSG criteria are important in the standardization of definitions used for IPA in clinical research studies, it does not demonstrate the true incidence of IPA in hematologic patients [8,9], and it does not include the non-culture-based diagnostic techniques. However, rapid and effective treatment of IPA is important because most patients will need subsequent chemotherapy for the underlying hematologic disease as soon as possible [10,11]. Given the lack of diagnostic certainty, and mortality and morbidity rate associated with IPA, empirical initiation of antifungal has been recommended for neutropenic patients with fever who do not respond to broad-spectrum antibiotics [5,10,12]. There are several studies indicating that antifungal treatment in combination with surgery improves survival in patients with acute leukemia, and lung tissue resection led to clearance of disease in 72% to 100% of patients [13]. A surgical procedure, excluding emergency operation for massive hemopthysis, is mainly used for either resection of a localized nodule or nodules that persist despite antifungal treatment or to prevent massive pulmonary hemorrhage that might be fatal or to eradicate residual fungal foci prior to further chemotherapy or BMT [3,14].

We report a case of acute promyelocytic leukemia that complicated with IPA due to *A. flavus *at relapse in 2003 10 years after initial diagnosis, and who was successfully treated with liposomal amphotericin B (L-AmB) by a combination of surgical resection. This report emphasizes the clinical picture and applicability of recent advances in the diagnostic and therapeutic approaches for IPA.

## Case presentation

**Figure 1:**
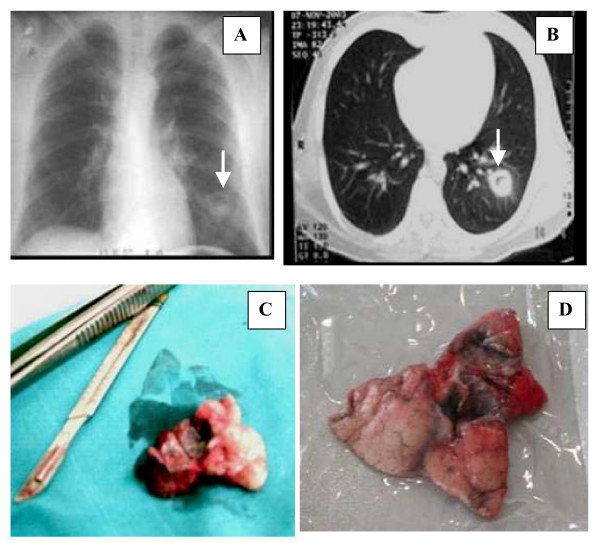
(**A**) Chest X-ray, (**B**) CT scan of the chest: a cavitary lesion showing an "air-crescent shadow" in the left lower lobe, (**C, D**) Resected pulmonary cavitary specimen without fungus ball.

Acute promyelocytic leukemia (AML-M_3_, APL) was diagnosed in a 33-year-old male in August 1993 who was successfully treated with doxorubicin and cytosine arabinoside (ARA-C). In July 2003, a second relapse of leukemia was established and the patient achieved complete hematologic remission following induction chemotherapy. He then received consolidation chemotherapy. In neutropenic period, he developed first neutropenic fever that resolved with broad spectrum antibiotics. But, fever recurred and he developed signs of respiratory failure with tachypnea. Chest X-ray showed pulmonary infiltration in the left lower lobe. Intravenous broad spectrum antibiotics were started, and on day 3 of the beginning of respiratory symptoms, intravenous L-AmB (1 mg/kg/day) was added as an antifungal therapy. *Aspergillus *was not cultured from his sputum, and galactomannan antigen (GM), (Platelia^® ^*Aspergillus*; Bio-Rad Laboratories, France) and blood culture were negative. Testing for *Aspergillus *antigenemia presented positivity and L-AmB dose was increased to 3 mg/kg/day, on day 6. At that time, high-resolution computed tomography (HRCT) showed the presence of pulmonary infiltrates in the left and right superior lobes and a cavity in the left lower lobe (Figure 1A,B). Thereafter, his respiratory condition improved and fever resolved within three days, and he recovered from neutropenia without use of granulocyte colony stimulating factor a week later. On day 11, a chest X-ray demonstrated reduction of pulmonary infiltrates, however the cavitary lesion persisted. After informed consent of the patient was obtained he underwent surgical treatment under general anesthesia on day 20. There was no adhesion in the thoracic cavity. The lesion was easily identified and pulmonary wedge resection was performed (Figure 1C,D). The operative course was uneventful and blood loss was minor. *Aspergillus flavus *was cultured and many *Aspergillus *hyphae were demonstrated within the surgical specimen. In January 2004, a third relapse of leukemia was established. He again underwent induction and further chemotherapies, and then, in February 2005, he received allogeneic stem-cell transplantation. These procedures were established without IPA reactivation and he had no side effect thought to be secondary to pulmonary resection. However, three months after stem-cell transplantation, he died due to intracerebral hemorrhage.

## Discussion

*Aspergilli *are ubiquitous in the environment and more than 180 species of *Aspergillus *have been identified; however only few are considered as pathogenetic in human beings, notably *A. fumigatus*, *A. flavus*, *A. niger*, *A. terreus *and *A. nidulans*. *Aspergillus fumigatus*, a conidium of 1–3 μm in diameter, is the most common frequent species found in 90% of infections. It is carried by air and overcomes the defensive abilities of upper respiratory tract and penetrates up to distal alveolar spaces. Thereafter, it germinates into angioinvasive filamentous hyphae that produce local tissue damage, hemorrhage, infarction and coagulative necrosis [6,15-17].

IPA generally presents as an acute infection. Sudden onset of shortness of breath, pleuritic chest pain, hemopthysis, pulmonary infiltrates, and fever un-responsive to broad-spectrum antibiotics constitute the characteristic clinical picture of the disease [15,16]. Early diagnosis of IPA remains difficult mainly because clinical signs and symptoms are not specific, culture and microscopy of lower respiratory tract specimens have low sensitivity, and histopathological examination of infected tissue is not easy due to often-poor condition and underlying coagulation abnormalities of patient [15]. Chest radiograph findings are not sensitive and specific for IPA, and also it may be normal in up to 10% [6]. HRCT scan is the most sensitive radiological method to detect early changes of IPA. "Halo sign" which correlates with hemorrhage and edema surrounding an infarct by thrombosis occurs early in the course of infection and is highly suggestive of IPA in patients with neutropenia and leukemia [6,15-17]. Air crescent sign, which is a central necrotic nodule with circumferential air density, develops mainly at the time of BM recovery. A cavitary lesion is the late stage of IPA. But, both halo sign and air crescent sign are not pathognomonic for aspergillosis and they can also be found in other fungal, as well as bacterial infections and other pulmonary disorders [12,15,17]. Recently, because of the difficulties in diagnosis of IPA, the European Organization for Research and Treatment of Cancer (EORTC) and Invasive Fungal Infections Cooperative Group and the Mycoses Study Group (MSG) of the National Institute of Allergy and Infectious Disease established consensus definitions for defining opportunistic invasive fungal infections based on a combination of host factors, clinical manifestations, and mycological results (Table 1) [7]. However, recently, Subira et al [8] studied the clinical applicability of EORTC/MSG classification in 22 patients with hematological malignancies who had IPA at autopsy and found that while alive, according to the EORTC/MSG criteria, only 2 patients had proven IPA, 6 probable, 13 possible and 1 was unclassifiable.

**Table 1:** EORTC/MNG criteria for definition of IPA

**Type of IPA**	**Criteria**
Proven	Demonstration of fungus in tissue histopathology or positive culture of tissue obtained by invasive procedure
Probable	One host factor plus one clinical feature plus one mycological factor
Possible	one host factor plus two minor clinical feature or one major clinical factor

One noninvasive tool currently applied to the diagnosis of invasive aspergillosis (IA) is GM, a major aspergillar cell-wall constituent, which is released during invasive disease. Serial screening for GM, complemented by appropriate radiologic techniques, can help to establish an early diagnosis of IPA in neutropenic patients with leukemia. Antigenemia can precede the diagnosis of IA by 8 and 9 days in 80% and 88.8% of patients, respectively. However, the optimal threshold for positivity remains a matter of debate [12,18]. Furthermore, there are still several problems in its applicability because of false-positive and false-negative reactions reported [15]. The case that we presented was a patient with high-risk acute myeloid leukemia (AML-M_3_, or APL) who underwent chemotherapy due to a second relapse after 10 years following diagnosis, with an occurrence of patchy pulmonary infiltrates and, subsequently, pulmonary cavitation due to *A. flavus *infection during consolidation therapy. We actually believe that our patient is a well case of IPA regarding difficulties in diagnosis of IPA, negative clinical applicability of EORTC/MSG criteria, and both positive and false-negative applicability of *Aspergillus *antigenemia in practice. Our patient can be classified as possible IPA according to the EORTC/MSG criteria, but it was demonstrated that he had true IPA. The patient was under the *Aspergillus *antigen testing for two times per week from the day of deep neutropenia; however GM positivity (cut-off index ≥ 1) was observed six days after the beginning of respiratory symptoms. The negativity of antigenemia might be due to the use of empirical L-AmB therapy. Nonetheless, GM index ≥ 1 warranted us to increase the dose of anti-*Aspergillus *therapy. Because we could not perform early HRCT evaluation, we did not observe halo sing in our patient. We observed a cavitary lesion showing an "air-crescent shadow" in the left lower lobe, which was the sign of BM recovery.

The optimal therapeutic management of IPA is controversial, ranging from different antifungal drugs to additional lung resection. Amphotericin B deoxycholate (AmB) has been used for many years as drug of choice for the treatment of IPA. The newer therapeutic options include: a) lipid formulations of AmB; b) azole antifungals, with anti-*Aspergillus *activity, itraconazole, and the third generation azole drugs, voriconazole, ravuconazole, posaconazole; and c) caspofungin, which belongs to a new class of antifungals, the echinocandines. Response to antifungal therapy in patients with acute leukemia complicated with IPA is highly individualized and depends on immune status, extent of disease and tolerability of drugs, and the prognosis is poor if the neutrophile count does not recover [15,19]. In our patient, we administered L-AmB at a dose of 1 mg/kg/day because of negative criteria for IPA. L-AmB is the only true liposomal formulation and admitted empiric treatment for febrile neutropenia resisting antibiotics as it has been tested in various clinical trials and proved to be effective and safe [20-23]. Although, Ellis et al [24] in an EORTC international multicenter randomized trial (EORTC number 19923) in which neutropenic patients with cancer or those undergoing BMT compared two dosages of L-AmB (1 mg/kg/d versus 4 mg/kg/d) found that a 1 mg/kg/d dosage was as effective as 4 mg/kg/d dosage, generally the recommended dose regimen of L-AmB is 3–5 mg/kg/d [25]. In our case, L-AmB which was increased to the dose of 3 mg/kg/d when the GM test became positive was found to be effective for IPA due to *A. flavus*. We were not able to administer one of the recent molecules (voriconazole or caspofungin), because in 2003 these drugs were not licensed in our country. New antifungal agents (voriconazole and caspofungin) were found to lead to better responses and improved survival and fewer side effects when compared with AmB as an initial therapy [26,27]. In several studies voriconazole and caspofungin were compared with L-AmB as empirical antifungal therapy in patients with febrile neutropenia, and it was shown that these drugs were non-inferior to L-AmB [22,26-28].

Administration of antifungal therapy and recovery of circulating neutrophils limit the infection, which then follows a subacute clinical course. At this stage, the involved area may cavitate and localize to a smaller, more discrete area [29]. *Aspergillus *has a characteristic tropism for the vascular wall and with marrow recovery; white blood cells lead to necrosis and increase the risk of hemorrhage by arterial perforation [3]. Also, the persisted cavitary lesion (as in our case) requires administration of anti-fungal therapy for several weeks before it disappears. So, surgical resection of the cavitary lesion may be necessary if treatment of the acute leukemia requires another chemotherapeutic regimen because of the high-risk of reactivation of Aspergillosis and massive hemoptysis with likelihood of death [2,3,19,30-33]. In this setting, a lobectomy, a segmentectomy, or a wedge resection, depending on the location and size of the aspergillosis lesion may be required [3]. In our patient, the clinical course evolved into a subacute form of aspergillosis due to neutrophile recovery and possibly, as a result of promptly initiated antifungal therapy. Because of the necessity for continuing therapy against leukemia, as well as the difficulty of eradicating aspergillosis in cavitary lesions and preventing aspergillosis reactivation, the solitary lesion in his left lung was resected. The surgical treatment was performed at 20^th ^day, because previous condition of the patient was not suitable for operation due to coagulation abnormalities. We did not observe an infectious, hemorrhagic or another complication related to general anesthesia and surgical intervention. This procedure made consecutive therapies safe and timely.

In conclusion, for early identification of a patient infected with IPA, a high index of suspicion and careful clinical and early radiological examinations with serial screening for GM should be established, and prompt administration of antifungal agents to improve the outcome of IPA should be considered. Finally, the combination of antifungal agents with surgical resection is an efficient and may be a cost effective strategy to eliminate residual pulmonary lesions for the treatment of IPA and to permit completion of therapies in patients with acute leukemia.

## Competing interests

The author(s) declare that they have no competing interests.

## Authors' contributions

RA coordinated and participated in design and in the drafting of the manuscript. FO and TO participated in its design and helped draft of the manuscript. VO and AO participated in the acquisition of data and management of the patient. AU, GO and AT participated in the management of the patient. SB participated in the surgical procedure. BE participated in laboratory data collection and evaluation. All authors read and approved the final manuscript.
